# Full-Genome Sequencing as a Basis for Molecular Epidemiology Studies of Bluetongue Virus in India

**DOI:** 10.1371/journal.pone.0131257

**Published:** 2015-06-29

**Authors:** Sushila Maan, Narender S. Maan, Manjunatha N. Belaganahalli, Pavuluri Panduranga Rao, Karam Pal Singh, Divakar Hemadri, Kalyani Putty, Aman Kumar, Kanisht Batra, Yadlapati Krishnajyothi, Bharat S. Chandel, G. Hanmanth Reddy, Kyriaki Nomikou, Yella Narasimha Reddy, Houssam Attoui, Nagendra R. Hegde, Peter P. C. Mertens

**Affiliations:** 1 Vector-borne Viral Diseases Programme, The Pirbright Institute, Ash Road, Pirbright, Woking, Surrey, GU24 0NF, United Kingdom; 2 College of Veterinary Sciences, LLR University of Veterinary and Animal Sciences, Hisar, 125 004, Haryana, India; 3 Ella Foundation, Genome Valley Hyderabad, 500 078, T.S, India; 4 Pathology Laboratory, Centre for Animal Disease Research and Diagnosis, Indian Veterinary Research Institute, Izatnagar, 243122, U.P, India; 5 National Institute of Veterinary Epidemiology and Disease Informatics (NIVEDI), Hebbal, Bengaluru, 560024, K.A, India; 6 College of Veterinary Science, Acharya N.G. Ranga Agricultural University, Rajendra Nagar, Hyderabad, 500 030, T.S, India; 7 Veterinary Biological & Research Institute, Govt. of Andhra Pradesh, Hyderabad, 500028, T.S, India; 8 College of Veterinary Science and AH, S.D. Agricultural University, Sardarkrushinagar-385 506, B.K., Gujarat, India; Rega Institute for Medical Research, BELGIUM

## Abstract

Since 1998 there have been significant changes in the global distribution of bluetongue virus (BTV). Ten previously exotic BTV serotypes have been detected in Europe, causing severe disease outbreaks in naïve ruminant populations. Previously exotic BTV serotypes were also identified in the USA, Israel, Australia and India. BTV is transmitted by biting midges (*Culicoides spp*.) and changes in the distribution of vector species, climate change, increased international travel and trade are thought to have contributed to these events. Thirteen BTV serotypes have been isolated in India since first reports of the disease in the country during 1964. Efficient methods for preparation of viral dsRNA and cDNA synthesis, have facilitated full-genome sequencing of BTV strains from the region. These studies introduce a new approach for BTV characterization, based on full-genome sequencing and phylogenetic analyses, facilitating the identification of BTV serotype, topotype and reassortant strains. Phylogenetic analyses show that most of the equivalent genome-segments of Indian BTV strains are closely related, clustering within a major eastern BTV ‘topotype’. However, genome-segment 5 (Seg-5) encoding NS1, from multiple post 1982 Indian isolates, originated from a western BTV topotype. All ten genome-segments of BTV-2 isolates (IND2003/01, IND2003/02 and IND2003/03) are closely related (>99% identity) to a South African BTV-2 vaccine-strain (western topotype). Similarly BTV-10 isolates (IND2003/06; IND2005/04) show >99% identity in all genome segments, to the prototype BTV-10 (CA-8) strain from the USA. These data suggest repeated introductions of western BTV field and/or vaccine-strains into India, potentially linked to animal or vector-insect movements, or unauthorised use of ‘live’ South African or American BTV-vaccines in the country. The data presented will help improve nucleic acid based diagnostics for Indian serotypes/topotypes, as part of control strategies.

## Introduction

Bluetongue (BT) is a non-contagious arboviral disease of domestic and wild ruminants that is transmitted via the bites of adult females of certain *Culicoides spp*. (Diptera, Ceratopogonidae) [1–5]. Bluetongue virus (BTV) is occasionally also transmitted ‘vertically’ across the placenta, in seminal fluid, by an oral route [6, 7], or directly through direct contact [8].

BT is characterized by oedema of the head and neck, inflammation of the muzzle area, ulcers around the teeth and on the tongue, rapid weight loss, depression, diarrhoea, inflammation in the coronary band and laminar corium of the hoof resulting in lameness. Severe cases may be fatal, particularly in sheep and some species of deer [9, 10]. The disease is more severe in naïve ruminant populations, or after introduction of an exotic strain/topotype to a new region [11–13].

*Bluetongue virus* is the type species of the genus *Orbivirus*, family *Reoviridae*. The BTV genome is composed of ten linear segments of dsRNA (Seg-1 to Seg-10 in order of decreasing molecular weight), packaged within a triple layered icosahedral protein capsid that is approximately 90 nm in diameter [14–17]. The BTV genome segments encode seven structural proteins (VP1 to VP7) and four non-structural proteins (NS1, NS2, NS3/NS3a, and NS4). BTV is closely related to several other economically important orbiviruses, including African horse sickness virus (AHSV) and Epizootic haemorrhagic disease virus (EHDV), with which it shares physico-chemical characteristics and vector species, although differing in host range [2].

Genetic diversity in the segmented genome viruses (including BTV can be generated by mutation, as well as by high frequency exchange of genome segments during co-infections (reassortment), leading to evolution of strains under strong purifying selection [18]. The spread and co-circulation of new BTV strains or serotypes into a region, facilitates reassortment and increases the likelihood of novel reassortant-strains being generated and emerging [19–21]. Intra-segment recombination and concatermerisation have also been described as mechanisms generating genetic diversity in BTV or related orbiviruses, although these appear to be less frequent events [22–24].

The majority of the BTV structural and non-structural proteins are highly conserved, including: VP3(T2) [901 aa inner, sub-core-shell protein]; the three minor enzymatic proteins, which include VP1 [RNA dependent RNA polymerase, 1302 aa], VP4 [capping enzyme and transmethylase, 644 aa] and VP6 [RNA dependent ATPase and helicase, 329 aa]); non-structural proteins NS1 [tubule forming protein, 553 aa]; NS2 [viral inclusion-body protein, 357 aa]; and the recently identified NS4 protein [aa 77] [25, 26]. This conservation reflects important structure-function constraints during virus replication and assembly. However, as exemplified by VP3, these proteins do show sequence variations that correlate with their geographic origins and can be used to group them as distinct ‘topotypes’ [27–29]. The aa and nt sequences of VP3/Seg-3 form three distinct major-topotypes, eastern, far-eastern and western, as well as ‘minor’ sub-groups (e.g the American and African viruses respectively, within the major western topotype). Evidence of further major topotypes of Seg-3 (although from few representative isolates), is provided by the Australian BTV-15 (strain DPP192), BTV-25 SWI2008/01 from Switzerland and BTV-26 from Kuwait KUW2010/01, each of which groups separately [30].

Seg-10 encodes non-structural proteins NS3 (229 aa) and NS3a (216 aa), which have been shown to play a role if virus-exit from infected cells [31–34]. This segment is highly conserved with sequence identity >75.9% nt / 81.2% aa across all of BTV isolates, but separates into three major groups–western-topotype 1 and 2 and eastern-topotype 1 [28, 30]. Two additional eastern-topotypes (E2 and E3) were previously proposed but to the date they contain only a single virus sequence each–BTV-15 China strain V447 and BTV-26 KUW2010/02 respectively. One additional western topotype (W3) has been proposed, containing BTV-25 (SWI2008/01).

The BTV outer-core protein VP7(T13) (349 aa) is encoded by Seg-7 [16]. Although VP7 is highly conserved and represents an immunodominant bluetongue-specific antigen, it splits into four distinct eastern and seven distinct western groups [28]. VP7 can mediate infection of insect vector cells, and it has been suggested that these groups could reflect interactions with distinct insect-vector populations [35–38].

The outer-capsid of the BTV particle is composed of 60 trimers of VP2 (OC1) and 120 trimers of VP5 (OC2), encoded by the most variable regions of the BTV genome, Seg-2 and Seg-6 respectively. The high degree of variability seen in these orbivirus outer-capsid proteins is thought to reflect the influence of antibody selective pressure in the vertebrate host. VP2 [950–962 aa in length] represents a primary target for neutralising antibodies and consequently its sequence determines virus serotype [30, 39]. Although Seg-6 and VP5(OC2) [526–527 aa] also show high levels of sequence variations, they are more conserved than Seg-2/VP2, and their variation does not show an absolute correlation with serotype. Some BTV strains have been identified with similar versions of Seg-6 belonging to distinct serotypes, while distinct versions of Seg-6 have been detected in the isolates of same serotype [40–43]. However, within a single BTV serotype, Seg-2 and Seg-6 also show variations that reflect the geographic origins of the virus isolate / genome segment, that clearly identify distinct ‘topotypes’, for example the major eastern- and western-topotypes previously identified by Maan et al [28, 44].

There are twenty seven known BTV serotypes, the global distribution of which has altered in recent years, possibly in response to changing climate, travel and trade [30, 45–49], There are two further putative/novel BTV serotypes: BTV-28 detected in a Capripox vaccine preparation in the Middle East (Kyriaki Nomikou, personal communication), and BTV-29, isolated from a llama in South Africa [50].

BT was first reported on the Indian subcontinent, in Pakistan, as early as 1958 [51] and subsequently in Maharashtra state in 1964 [52]. Since then outbreaks of BT have been reported from several other regions of the subcontinent and the virus is now considered to be endemic in both India and Pakistan [53, 54]. However, there have also been reports concerning the incidence and prevalence of BT in other countries in the region, including Afghanistan, Bangladesh, Nepal, Bhutan, Mongolia and Sri Lanka [55, 56].

BT has a very significant impact on livestock in India. Circulation of the virus represents a significant constraint both in the rearing and maintenance of indigenous sheep breeds, which often exhibit severe clinical disease, as well as in the use of improved breeds, which have the potential to raise overall productivity. There are no detailed estimates of the economic losses caused by BTV on the subcontinent, although family-based, subsistence farming communities living within rain-fed agricultural systems in the south of the country are particularly severely affected. In addition to consistent yearly losses, periodic monsoon-driven hyper-endemic outbreaks of BTV can kill hundreds of thousands of sheep.

Vaccination is being used to control BTV in India. A pentavalent inactivated vaccine containing BTV serotypes 1, 2, 10, 16 and 23 is being marketed by Biovet and Indian Immunologicals, although so far only sheep are being vaccinated. Vaccination needs to be done one month before the onset of monsoon and efficacy of vaccination is being tested in Tamil Nadu, Andhra Pradesh, Karnataka and Maharashtra states.

The BTV episystem of the Indian subcontinent supports circulation of the majority of BTV serotypes. However, there is lack of systematic studies concerning the characterisation and spatial distribution of BTV strains, genotypes, serotypes and topotypes in the region. Genotype/topotype analysis of Indian BTV isolates has previously relied upon comparisons of sequence data for selected genome segments derived from different isolates, rather than complete genome analyses [57–59]. While the data generated have provided some insights into the relationships and likely origins of the BTV strains circulating in India, the lack of a comprehensive strategy for determining the sequence of all genome-segments of multiple isolates, means that the geographic movement and ‘flow’ of individual genes associated with reassortment is still difficult to detect and interpret. Full-genome sequence data have recently been generated for multiple BTV isolates from India, providing information concerning the circulation of both eastern and western-topotypes, as well as identifying vaccine and reassortant strains [60–65].

This paper describes the use of simple and reliable methods for full-genome sequence analyses of multiple BTV isolates from India. It provides a comprehensive analysis of representative strains of different Indian BTV serotypes, revealing their relationships to those from rest of the world and providing evidence for multiple entries of different serotypes and exotic topotypes into India in recent times.

## Materials and Methods

### Virus propagation in cell culture

Isolates of bluetongue virus were obtained from the Orbivirus Reference Collection (ORC) at The Pirbright Institute (TPI). These samples were not collected for research purposes, but were taken from naturally infected/dead animals in the field, by qualified veterinarians, as part of normal diagnostic testing procedures. Individual strains are identified by a unique reference collection number, composed of ‘country code’, year, and the number of the isolate in that year from that country. Full details of these isolates are available on the dsRNA virus reference collection website (http://www.reoviridae.org/dsRNA_virus_proteins/ReoID/BTV-Nos.htm) and associated GenBank entries are listed in S1 Table.

The viruses were propagated in BHK-21 clone 13 cells (European Collection of Animal cell Cultures [ECACC– 84100501]) at 37°C in Dulbecco’s minimum essential medium (DMEM) supplemented with antibiotics (100 units/ml penicillin and 100 mg/ml streptomycin) and 2 mM glutamine. Infected cell cultures (175 cm^2^ culture flask per sample at 70% to 80% cell confluence) were incubated at 37°C, until they show characteristic and widespread (>70%) cytopathic effects (CPE), usually between 48–72 hours post infection. The viruses were harvested 3 to 4 days post-infection, aliquoted and used for viral dsRNA extraction, or stored in the ORC at minus 80°C.

### Preparation of BTV dsRNA

Intact genomic dsRNA was extracted from BTV infected cell cultures using a guanidinium isothiocyanate extraction procedure, as described earlier by Attoui et al [66]. The infected cell pellet was lysed in 1 ml of commercially available TRIZOL reagent (Invitrogen) and total RNA extracted. dsRNA was prepared by differential precipitation of ssRNA using 2M LiCl and recovery of the dsRNA from the supernatant by precipitation with isopropanol. The integrity of the dsRNA was assessed after separation on a 1% agarose gel in 1X Tris acetate EDTA (TAE) buffer containing 0.5μg/mL of ethidium bromide.

### Reverse transcription of dsRNA, PCR amplification and sequencing of cDNA segments

BTV genome segments were reverse-transcribed into cDNA using full-length amplification of cDNAs (FLAC) as described by Maan et al [67]. Briefly, a 35 base oligonucleotide ‘anchor-primer’, with a phosphorylated 5’ terminus, was ligated to 3’ ends of the viral dsRNAs using T4 RNA ligase, followed by reverse transcription using ‘RT system’ (Promega). The resulting cDNAs were amplified using primers complementary to the anchor primer. For sequencing purposes, a high fidelity KOD polymerase enzyme (Novagen) was used in the PCR. These methods produced from 100 ng to 1000 ng of BTV cDNA per 175 cm^2^ flask of infected cells that was of a quality suitable for the ABI 3730 sequencing platform. The sizes of the resulting cDNA amplicons were analyzed and confirmed by agarose gel electrophoresis. Individual cDNA amplicons, purified using a ‘GFX PCR DNA and gel band purification kit’ (Amersham Pharmacia Biotech, Inc), were sequenced on a 3730 capillary sequencer (Applied Biosystems). ‘Phased primers’ [67] were used to sequence the near-terminal regions of all ten genome segments. The full-length sequences of individual genome segments were completed by primer-walking using segment-specific primers. The inclusion of primers (both inward and outward facing) targeting the conserved 5’ and 3’ ends of the BTV genome segments, improved the low coverage routinely observed towards the ends of the genome-segments (data not shown). Data generated for the full-genomes of prototype BTV strains, belonging to different serotypes from India, have been submitted to GenBank (S1 Table).

### Sequence analysis and phylogenetic tree construction

‘Raw’ ABI sequence data was assembled into ‘contigs’ using the SeqManII sequence analysis package (Lasergene version 5). Additional BTV sequences were downloaded from GenBank as on 6^th^ December 2014. The open reading frames (ORFs) of BTV genome segments were identified using EditSeq software (implied within Lasergene version 5) and NCBI ORF finder (http://www.ncbi.nlm.nih.gov/gorf/gorf.html), and translated to amino acid (aa) sequences for further analysis. Multiple alignments of consensus sequences were performed using Clustal X (Version 2.0) [68], Clustal Omega [69] and MAFFT [70], to ensure proper alignment. RevTrans 1.4 Server was also used to translate nucleotide data, which aligns the resulting amino acid sequences, then uses this ‘Platform’ to construct nucleotide alignments that maintain reading frame integrity [71]. Pairwise distance (aa and nt) calculations and phylogenetic trees constructions were done using MEGA 5 software [72] using the p-distance parameter, neighbor-joining method and tested by bootstrapping 1000 replicates [73]. MEGA was used to find the best-fit substitution model for each genome segment. The model with the lowest Bayesian Information Criterion (BIC) was used to undertake a Maximum Likelihood analysis with 1000 bootstraps. The nomenclature for the geographically based phylogenetic groups (topotypes) is as described by Maan et al [28, 44].

## Results

### Sequence and phylogenetic analysis of BTV genome segments

**Fig 1:**
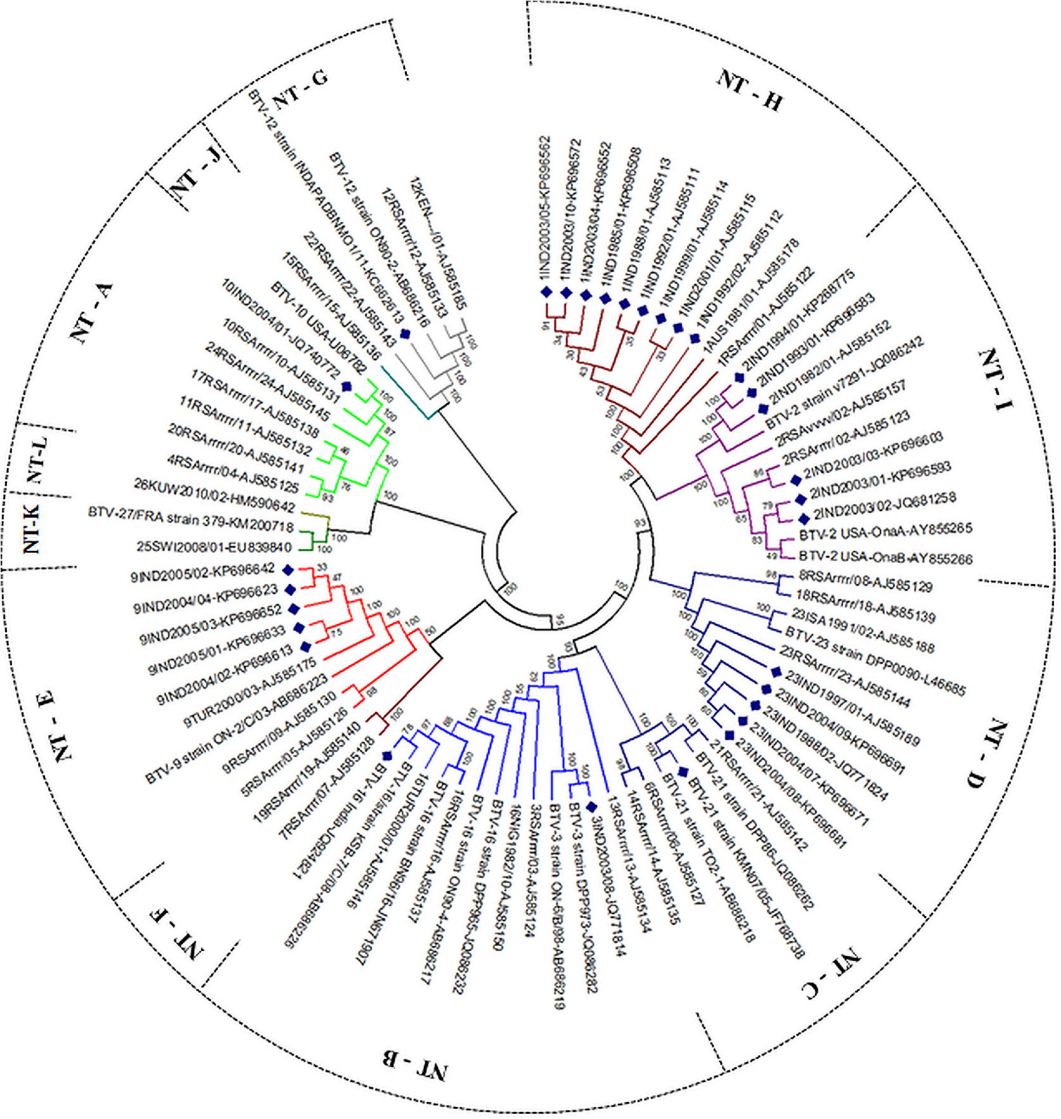
Phylogenetic analysis based on Seg-2/VP2 gene of Indian isolate of BTV with other global isolates. Phylogenetic relationship of full length Seg-2 nucleotide sequences (n = 80) was inferred in MEGA 5 using neighbour-joining method and tested by bootstrapping 1000 replicates. Seg-2 nucleotypes were assigned as per Maan et al [28, 30] and depicted by different branch colour. Indian isolates are depicted with blue dots. The node labels in each figure refer to bootstrap confidence values.

**Fig 2:**
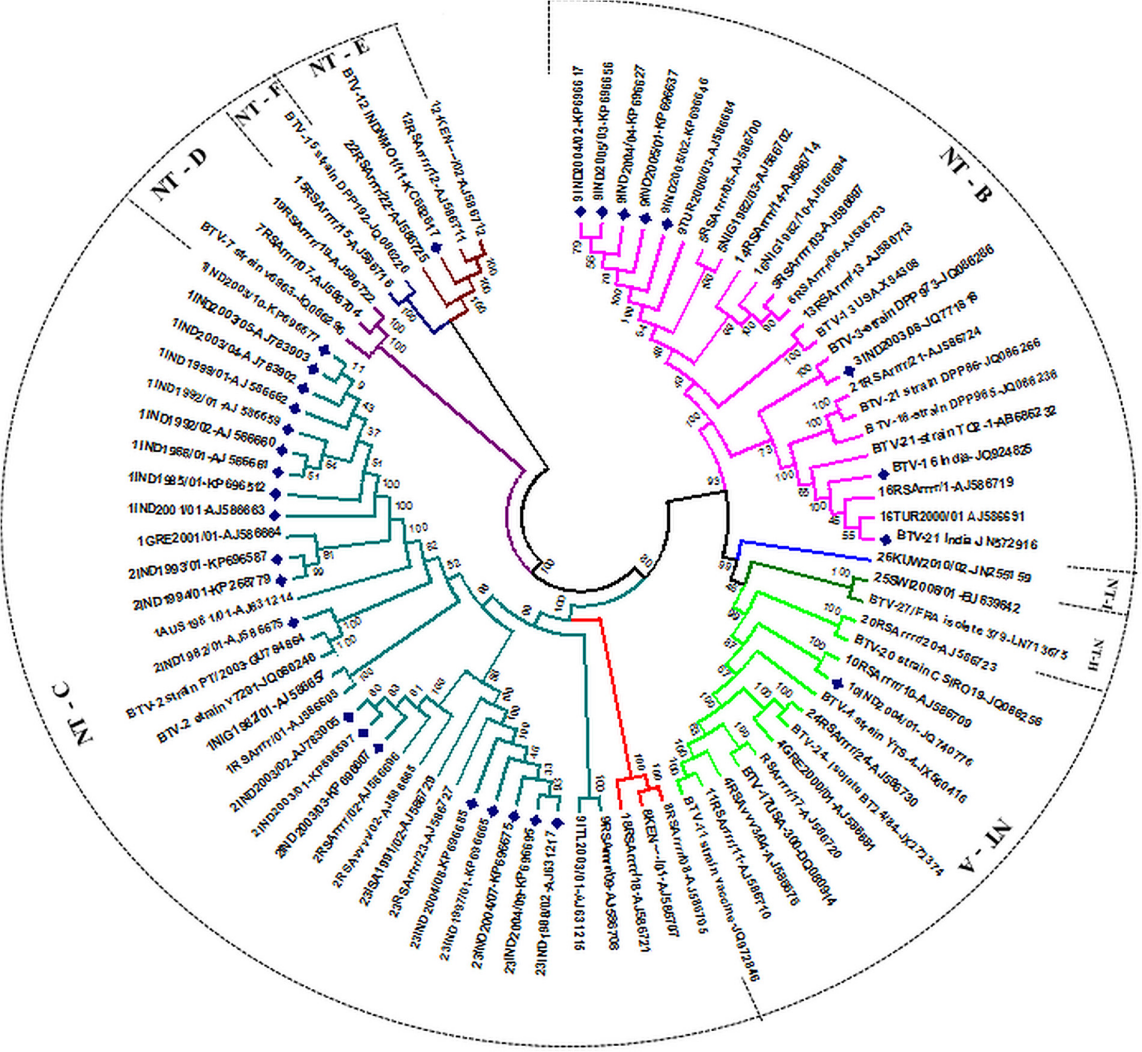
Phylogenetic analysis based on Seg-6/VP5 gene of Indian isolate of BTV with other global isolates. Phylogenetic relationship of full length Seg-6 nucleotide sequences (n = 84) was inferred in MEGA 5 using neighbour-joining method and tested by bootstrapping 1000 replicates. Seg-6 nucleotypes were assigned as per Maan et al [28, 30] and depicted by different branch colour. Indian isolates are depicted with blue dots.

**Fig 3:**
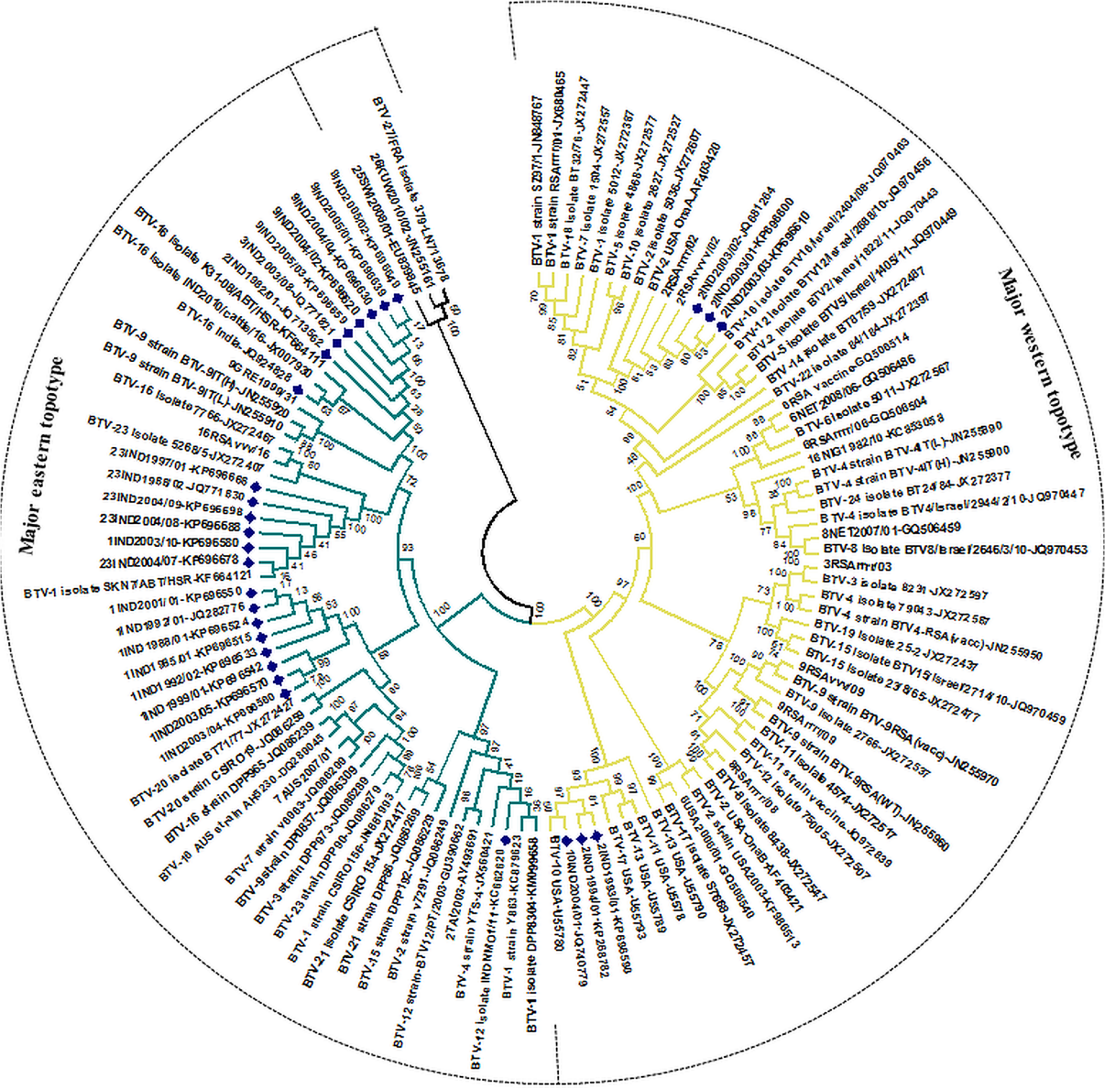
Phylogenetic analysis based on Seg-9/VP6 gene of Indian isolate of BTV with other global isolates. Phylogenetic relationship of full length Seg-9 nucleotide sequences (n = 114) was inferred in MEGA 5 using neighbour-joining method and tested by bootstrapping 1000 replicates. Topotypes were assigned as per Maan et al [28, 30] and depicted by different branch colour. Indian isolates are depicted with blue dots.

**Fig 4:**
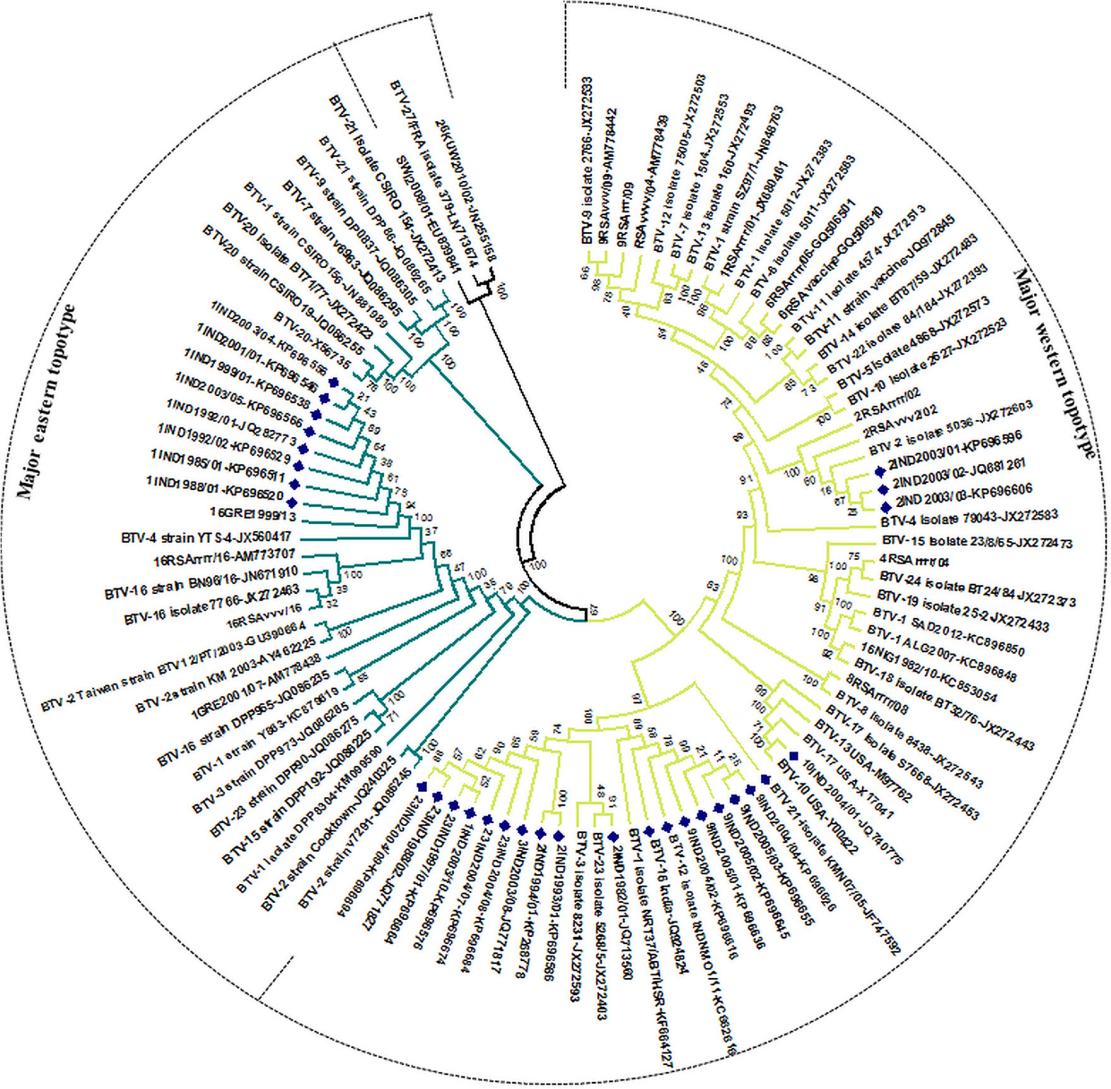
Phylogenetic analysis based on Seg-5/NS1 gene of Indian isolate of BTV with other global isolates. Phylogenetic relationship of full length Seg-5 nucleotide sequences (n = 98) was inferred in MEGA 5 using neighbour-joining method and tested by bootstrapping 1000 replicates. Topotypes were assigned as per Maan et al [28, 30] and depicted by different branch colour. Indian isolates are depicted with blue dots.

Full-length, full-genome nucleotide sequences were determined for twenty seven Indian BTV isolates (S1 Table). These data were used in ‘nearest neighbour analyses’, to identify the virus serotype (controlled by Seg-2/VP2) and the topotype of each genome segment, by comparisons to an existing sequence dataset for reference strains of each BTV serotype. Nucleotide substitution models obtained for different BTV genome-segment, using Bayesian Information Criterion (BIC), were: TN93+G+I (Seg-1, -2, -6 and -8); T92+G+I (Seg-3, -4 and -5) and T92+G (Seg-7); GTR+G+I (Seg-9) and GTR+G (Seg-10). The use of neighbour-joining (p distance), codon partitioning, and maximum likelihood methods did not significantly alter the clustering or phylogenetic relationships of any genome-segment (Figs 1–4).

### Outer-capsid genes/proteins

#### Seg-2/VP2(OC1)

Analysis of twenty-seven nucleotide sequences for Seg-2 (encoding VP2(OC1)) from Indian prototype strains of BTV, showed 69% nt sequence identity with Seg-2 from reference strains of the homologous BTV serotypes (S2 Table). This confirmed their serotype identifications. Seg-2 from Indian isolates of four serotypes (BTV-1 [IND1988/01], BTV-3 [IND2003/08], BTV-9 [IND2005/01], and BTV-23 [IND1988/02]), as well as previously published sequence data for Indian isolates of three serotypes (BTV-12 [Acc. No. KC662613; BTV-16 [Acc. No. JQ924821 and BTV-21 [Acc. No. JF768738] [43, 57, 74]), all showed >90% nucleotide identity with eastern strains of the same serotype, identifying them as ‘eastern topotype’ for Seg-2 (Fig 1). In each case Seg-2 also showed <75% identity to previously characterised ‘western’ strains of the same BTV serotypes. However, both eastern and western-topotypes of Seg-2 were identified for Indian strains of BTV-2, with the earliest strains [IND1982/01, IND1993/01, IND1994/01] representing an eastern topotype (BTV-2e), while the later isolates [IND2003/01, IND2003/02 and IND2003/03] representing a western topotype (BTV-2w). Seg-2 from these western strains [IND2003/01, IND2003/02 and IND2003/03] shared 99.7% nt identity with a vaccine strain of BTV-2 from South Africa [RSAvvvv/02]. The Seg-2 sequence generated for Indian isolates IND2004/01, IND2003/06 & IND2005/04], grouped within the ‘western’ topotype of BTV-10, showing 99.9% nt identity with BTV-10 (CA-8) [Acc. no. U06782] that was previously been used to generate a modified live BTV-10 vaccine [75] (S2 Table).

#### Seg-6/VP5(OC2)

Nucleotide sequences for Seg-6 (encoding VP5(OCP2)) from the 27 Indian BTV strains, were compared to previously published data from other BTV isolates. As seen for Seg-2, the same isolates of four serotypes (BTV-1 [IND1988/01], BTV-3 [IND2003/08], BTV-9 [IND2005/01] and BTV-23 [IND1988/02]), as well as BTV-16 [Acc. No. JQ924825 and JN572918] and BTV-21 [Acc. No. JN572916] [76], all grouped as ‘eastern-topotype’ with >77.1% nucleotide identity to previously characterised eastern isolates of the homologous serotypes. These isolates also showed < 80.1% nt identity with western strains of the homologous serotypes (Fig 2 & S3 Table). Two southern Indian isolates IND1993/01 and IND1994/01 of BTV-2 had Seg-6 that is identical to Indian BTV-1 strains [IND2003/05], demonstrating reassortment between their two outer-capsid protein genes. However, consistent with the data obtained for Seg-2(VP2), both eastern and western-topotypes of Seg-6 were identified for Indian strains of BTV-2. The earliest isolate [IND1982/01] contains an eastern Seg-6, while IND2003/01, IND2003/02 and IND2003/03 all contain a western topotype version of this segment with 99.9% nt identity to reference strain of BTV-2 (RSArrrr/02) (S3 Table). Seg-6 of BTV-10 [IND2004/01, IND2003/06 & IND2005/04] and BTV-12 [Acc. No. KC662617] were also identified as western topotypes with 99% and 97.8% nt identities to the prototype U.S. strain (CA-8) of BTV-10 (which represents the original source of the U.S. vaccine) and to reference strain of BTV-12 (RSArrrr/12) respectively.

### BTV outer core protein genes: Seg-7/VP7(T13) and Seg-3/VP3(T2)

#### Seg-7/VP7(T13)

Seg-7 from Indian isolates belonging to three serotypes (BTV-1 [IND1988/01], BTV-2 [IND1982/01] and BTV-23 [IND1988/02]) grouped along with viruses from China, Taiwan and Australia, within ‘eastern-topotype 2’. In contrast Seg-7 of BTV-3 [IND2003/08], BTV-9 [IND2005/01], BTV-16 India [Acc. no. JQ924826] and BTV-21 [Acc. No. JF768739] clustered with viruses from China, Australia, Turkey and Europe, within eastern-topotype 3, as previously identified by Maan et al [28]. However the later BTV-2 isolates from India [IND2003/01, IND2003/02 and IND2003/03] all grouped closely with BTV-2 USA (OnaA), isolates from Jamaica and South African reference and vaccine strains within ‘western topotype 2’ of Seg-7 (with >99.5% nt identities) (S1 Fig). Seg-7 from Indian isolates of BTV-10 [IND2004/01, IND2003/06 & IND2005/04] and that of a previously published isolate of BTV-12 from India [Acc. No. KC662618] grouped within ‘western-topotype 4’, along with viruses from the USA, Caribbean islands, Guatemala, Brazil, and Jamaica, with up to 99.8% and 99% nt identities between IND2005/04 and BTV-10 USA–[Acc. no. NC_006022]) and BTV-12 South Africa [Acc. No. JX272505], respectively.

#### Seg-3/VP3(T2)

The majority of the Indian isolates, (BTV-1 [IND1988/01], BTV-2 [IND1982/01], BTV-3 [IND2003/08], BTV-9 [IND2005/01], BTV-12 (Acc. No. KC662614), BTV-16 (Acc No. JQ924822) and BTV-23 [IND1988/02]) again grouped within the major eastern topotype for Seg-3 (>79% nt identity) (S2 Fig). However, Seg-3 of the later Indian BTV-2 isolates IND2003/01, IND2003/02, and IND2003/03 is identical to that of the South African reference and vaccine strains (RSArrrr/02 and RSAvvvv/02). It also groups very closely with BTV-2 USA (Ona A) (99% nt identity). Indian isolates of BTV-10 IND2003/06, IND2004/01 and IND2005/04 all grouped within the major western topotype for Seg-3, showing up to 100% nt identity with previous isolates of BTV-10, 11, 13 and 17 from USA (S2 Fig).

### BTV inner core protein genes: Seg-1 (VP1), Seg-4 (VP4) and Seg-9 (VP6)

#### Seg-1/VP1(Pol)

BTV Seg-1, encodes the highly conserved RNA polymerase VP1. Indian isolates IND1988/01 [BTV-1], IND1982/01 [BTV-2], IND2003/08 [BTV-3], IND2004/02 [BTV-9], BTV-12 (Acc. No. KC662612), BTV-16 [Acc. No. JQ924820], and IND1988/02 [BTV-23], all group closely (>80% identity) within the major eastern topotype for Seg-1. All of the Indian BTV-1 isolates analysed have an identical Seg-1, sequence except for IND2003/10, which falls into a separate subclade with 100% nt identity to Indian strains of BTV-23 (S3 Fig). The later BTV-2 isolates, IND2003/01, IND2003/02, and IND2003/03 have Seg-1 that is identical to that of the South African BTV-2 vaccine strain within the major western topotype, indicating a common origin. Similarly, Seg-1 of Indian BTV-10 isolates IND2003/06, IND2004/01 and IND2005/04, is identical to that of a BTV-10 strain from the USA (Accession nos. NC006023 and X12819), grouping with isolates and vaccine-like strains of BTV-10, 11, 13 and 17 from USA (S3 Fig).

#### Seg-4/VP4(CaP)

Seg-4 encodes the highly conserved RNA-capping enzyme VP4(CaP) which is a minor component of the core particle of BTV. Comparisons of Seg-4 generated results similar to those obtained with Seg-1 (see above), grouping the majority of Indian isolates regardless of serotype (including BTV-12 [Acc. No. KC662615]) within an eastern-topotype (>90 nt identity), with minor variations indicating sub-clades. Seg-4 of BTV-1 IND2003/05 is identical to that of BTV-9 isolates IND2005/01 and IND2005/02, while Seg-4 of BTV-1 IND2003/10 is identical to that of BTV-23, in both cases indicating common ancestries and the occurrence of reassortment events during the recent history of these isolates. The later Indian isolates of BTV-2 [IND2003/01, IND2003/02 and IND2003/03] showed 100% identity in Seg-4 with BTV-2 USA (OnaA), while Seg-4 of BTV-2 IND2003/02 is identical to that of the BTV-2w vaccine strain from South Africa (Accession no. JN255935), in both cases belonging to a western topotype. The Indian BTV-10 isolate [IND2004/01] showed up to 100% identity with other BTV-10 strains from USA (Accession nos. NC_006024 and Y00421) (S4 Fig).

#### Seg-9/VP6(Hel) & NS4

BTV Seg-9 encodes the conserved BTV helicase, VP6(Hel) and NS4 proteins [25, 26]. Seg-9 from majority of the Indian BTV isolates groups within an eastern topotype, irrespective of serotype, with minor variations indicating sub-clades, in a manner similar to Seg-1 and Seg-4 (Fig 3 and S4 Table). Segment 9 of a published BTV-12 strain from India [Acc. No. KC662620] also clusters with Asian/Australian isolates, but is more closely related to isolates from China, Taiwan and Australia than to Indian isolates (Fig 3 and S4 Table), indicating that this segment might have entered India recently. However, Seg-9 of some of earlier isolates of BTV-2 [IND1993/01 and IND1994/01] and later isolates of BTV-2 [IND2003/01, IND2003/02 and IND2003/03] grouped in the western cluster, very close to BTV-10(w) isolate USA/10O80Z [U55781] and BTV-10 [IND2004/01] (99% nt identities), suggesting a recent common western ancestry. Seg-9 of BTV-1 [IND2003/10] and the isolates of BTV-23 [e.g. IND1988/02 & IND1997/01], show 99.9% to 100% nt identities in Seg-9, indicating a recent common and eastern ancestry (Fig 3 and S4 Table).

### BTV non-structural protein genes: Seg-5 (NS1), Seg-8 (NS2) and Seg-10 (NS3)

#### Seg-5/NS1(TuP)

Seg-5 codes for non-structural protein NS1, which forms characteristic ‘tubules’ within BTV infected cells. All of the Indian of isolates of BTV serotype 1, (except IND2003/10), grouped within a major eastern-topotype for Seg-5, with 99–100% nt identity to a BTV-4 strain (YTS-4) from China and BTV-9 strains from Europe [BOS2002/01 & SER2001/01] (which also have an eastern origin) (Fig 4 and S5 Table). In contrast Seg-5 from the Indian isolates of BTV-2, 3, 9, 10, 12 [Acc. No. KC662616], 16 [Acc. No. JQ924824], 21 [Acc. No. JF747592], and 23 and BTV-1 [IND2003/10] all grouped together (with 99%/100% nt identity) within a major western topotype of Seg-5. Seg-5 of the BTV-2 [IND2003/01, IND2003/02 and IND2003/03] and BTV-10 strains [IND2004/01] like all other genome segments showed very high identities to the western vaccine strains (100%) (Fig 4 and S5 Table).

#### Seg-8/NS2(ViP)

Seg-8 encodes the highly conserved viral inclusion body matrix protein NS2 (354 aa). Analyses of Seg-8 sequences from the Indian BTV strains reinforced their separation into major eastern and western topotypes. Indian isolates of BTV-1 [IND1988/01], BTV-2 [IND1982/01], BTV-3 [IND2003/08], BTV-9 [IND2005/01], BTV-12 [Acc. No. KC662619], BTV-16 [Acc No. JQ924827], and BTV-23 [IND1988/02], all cluster within the major eastern topotype of Seg-8. In contrast Seg-8 from Indian isolates of BTV-2 [IND2003/01, IND2003/02, and IND2003/03] sits within the western topotype of Seg-8 with 99.7% nt identity to vaccine strains of BTV-2 from South Africa. The Indian BTV-10 isolates [IND2003/06, IND2004/01 and IND2005/04] grouped with isolates of BTV-10, 11, and 17 from the USA including the prototype U.S. strain (CA-8) of BTV-10 (with >99% nt identity) (S5 Fig).

#### Seg-10 /NS3

Seg-10 encodes non-structural glycosylated membrane proteins NS3 (229 aa) and its shorter form NS3A (216 aa), which are implicated in the exit of progeny visions from infected insect cells. Seg-10 can be divided into 3 eastern and 3 western subgroups [28, 30]. The Seg-10 sequences from Indian isolates of BTV-1 [IND1988/01], BTV-2 [IND1982/01], BTV-3 [IND2003/08], BTV-9 [IND2005/01], BTV-12 [Acc. No. KC662621], BTV-16 [Acc No. JQ924829], and BTV-23 [IND1988/02]) all group within an eastern-topotype subgroup 1, within different although closely related sub-clades. Seg-10 of BTV-9 IND2005/01 groups within western subgroup 1. Isolates of BTV-2 from India [IND2003/01, IND2003/02, IND2003/03] also grouped within western cluster 1, showing 99.9% nt sequence identity with South African vaccine strains of BTV-2 [Acc. no. JN255941]. Similarly, Indian strains of BTV-10 [IND2004/01, IND2003/06 and IND2005/04], which also grouped within western subgroup 1, showed 99.5% nt identity to BTV-10 USA (Acc. no. AF044381) (S6 Fig).

## Discussion

Sequencing of Seg-2/VP2 provides a reliable method to identify the serotype of novel BTV isolates. However, sequencing and phylogenetic analyses of the entire BTV genome can provide definitive information concerning the entire BTV genotype, identifying both the lineage and topotype of each individual genome-segment, in a manner that cannot be achieved by conventional serological diagnostic methods. The data generated by these phylogenetic analyses show both the lineage and topotype of each genome segment, identifying individual strains and reassortant strains and providing further information concerning their evolution and movements. The ‘resolution’ of these methods depends on both the quality of the sequence data generated, the availability of reliable data concerning the geographic and temporal origins of the individual strains/isolate analysed, as well as the availability of data for previously characterised strains that are held in a ‘reference’ data-set / virus collection.

Environmental changes can influence the incidence, distribution and evolution of infectious diseases, particularly those transmitted by arthropod vectors [77]. The global pattern of BTV serotype distribution has changed in recent years with multiple, previously exotic BTV strains and serotypes detected in Europe, North America, Australia, Korea, India, Middle east and South America [45, 47, 48, 78–83]. These events have been linked to changes in climate, increased travel and trade, and possibly to changes in land use or farming methods [45]. In order to be successfully transmitted, bluetongue virus must replicate in its *Culicoides* vectors, which are poikilothermic. Since the virion associated enzymes required for BTV replication have defined temperature optima in the region of 25^°^C to 42^°^C, transmission is more efficient at higher ambient temperatures and may be favoured by climate warming. The introduction of even small numbers of infected insects to previously ‘free’ areas is also thought to pose a significant risk of an outbreak. Since adult *Culicoides* could easily be transported accidentally by trade in a range of products, or as a result of international travel, these activities represent further risk factors. This suggests that an apparently stable pattern or absence of bluetongue disease in any region could be delicate and transient [45, 46, 84].

The development of better technologies and databases to identify and compare these viruses has recently led to the discovery and identification of multiple additional/novel serotypes (BTV-25, 26 and 27) [39, 49, 85].

Bluetongue has been reported in several parts of India, more frequently in the southern states of Andhra Pradesh, Karnataka and Tamil Nadu where it had been severe during 2004–2006. There is evidence for at least twenty two distinct serotypes on the sub-continent [86]. Thirteen BTV serotypes have been isolated in India (BTV-1, -2, -3, -4, -8, -9, -10, -12, -16, -17, -18, -21, -23), nine of these in the last decade [13, 87], with serological evidence for ten more (BTV-5, -6, -7, -11, -13, -14, -15, -19, -20, -24) [54, 57, 87, 88]. BTV-10 was reported for the first time in the east of India, in Kolkata during 2004, although there are no reports of BT in the north-eastern states [53, 54, 89].

The relationships (topologies) for the different genome segments of BTV isolates from different geographic locations around the world, are broadly very similar, with Seg-1, -3, -4, -8 and -9 falling into two major ‘eastern’ and ‘western’ topotypes [28]. BTV-15AUS/1982 is a notable exception, with multiple genome segments falling outside these topotypes. Recent isolates of BTV-25 and BTV-26 represent additional and more distantly related western and eastern groups/topotypes respectively in at least seven genome segments [30]. The evolutionary distance inferred for each genome segment between viruses representing these different eastern and western topotypes, indicates an extended period of geographically independent evolution [18, 30].

Most genome segments of the Indian BTV isolates of BTV-1, 2, 3, 9, 16, 21 and 23 that are analysed here, group within a major eastern-topotype [28]. However, Seg-5 of these Indian strains (with the exception of isolates of BTV-1, prior to 2003) group within the major western topotype, showing 97% nt identity with strains from Nigeria (NIG1982/01), Libya (LIB2008/03) and Oman (OMN2009/01). Majority of other genome segments in each case clearly belong to an eastern-topotype, indicating that that they originated from India or another part of eastern Asia. These analyses also show that in addition to Seg-5, Seg-9 of BTV-2 [IND1993/01 and IND1994/01] and Seg-10 of BTV-9 [IND2003/11] group within the western-topotypes (with 99% and 98.6% nt identity to BTV-10(w) isolates [USA/10O80Z - Acc. no. U55781 and USA/10B81U - AF044381] respectively).

In contrast, all genome segments of later Indian isolates of BTV-2 [IND2003/01, IND2003/02, and IND2003/03] show >99% identity with a vaccine strain of BTV-2 from South Africa. Indian isolates of BTV-10 [IND2003/06, IND2004/01 and IND2005/04] show >99% identity with the prototype strain of BTV-10 (CA-8) from the USA, which also provided the original source material for an attenuated BTV-10 vaccine [90, 91]. These BTV-2 and BTV-10 strains, are both clearly western topotype, and have not acquired genome segments by reassortment with the indigenous eastern / Indian strains, suggesting that they had only recently been introduced to the subcontinent.

The Central Sheep Breeding Farm (CSBF), Hisar, Haryana, India, imported Corriedale, Merino and Dorset sheep from Australia and Rambouillet sheep from America during late the 1970s and 1980s. Cattle were also imported into India during 2002 to 2005, from Belgium, France, Germany, Nepal, Russia, South Africa, Thailand, the UK and USA [13]. Some of these countries had used live attenuated vaccines during this period. These data suggest that the use of these live vaccines and international trade in livestock could both have played important roles in the introduction of western BTV strains into India. These exotic topotypes may persist, at least for a while, as intact ‘western’ genomes (as seen for BTV-2 and BTV-10), or in the longer term may contribute individual western genome segment(s) to the indigenous BTV gene pool (as seen with the western Seg-5).

It is also possible that despite trade restrictions, western topotype, live vaccine strains have been imported and used in India. Live attenuated BTV vaccines can cause significant levels of viraemia post vaccination, leading to infection of adult *Culicoides* during blood feeding, and onward virus-transmission [20, 92, 93]. They may also be more virulent in animals from regions that are distinct from their original geographic source (different topotype) [92].

High frequency reassortment of BTV genome segments, can occur in both vertebrate and invertebrate hosts, and the subsequent selection of the progeny strains leads to the emergence of novel BTV genotypes in the field. It appears likely that strains which ‘emerge’ will have a selective advantage over the other reassortants generated at the same time and may therefore replicate or be transmitted more efficiently. For example the dominant strain may replicate to higher titres in the ruminant hosts, enhancing infection rates and the efficiency of transmission by adult *Culicoides*, suggesting that they could also be more virulent. If a particular genotype confers a significant transmission advantage, it could potentially have been generated simultaneously in many different cells or even in different animals that are co-infected with the same combination of parental strains.

It is notable that a western topotype Seg-5 has spread relatively rapidly through the otherwise eastern topotype strains of BTV in India (since 1982). This rapid dissemination suggests that this version of Seg-5/NS1 significantly enhances the transmission of the otherwise eastern BTV strains in the region. Its dissemination also shows a temporal correlation with recent increases in virulence of BTV outbreaks in India [58, 61].

Full genome sequencing, followed by analysis of the topotypes and origins of individual genome segments, can provide evidence concerning the origins and evolution of individual reassortant viruses. Phylogenetic analyses for BTV strains in Europe and north Africa indicates that reassortment take place rapidly and progressively between co-circulating BTV strains, including the live attenuated vaccine strains used for disease control, leading to the emergence of novel genotypes (Nomikou, Biek et al.,—in preparation).

The existence of the majority of the BTV serotypes in the Indian Sub-continent would provide extensive opportunities for reassortment of novel BTV strains belonging to exotic topotypes. Phylogenetic analyses of the most prevalent Indian serotypes have identified multiple BTV strains that contain specific segments that are identical (or nearly identical) indicating a common origin and genome segment reassortment. The process of reassortment and selection of common genome segments may represent an important component mechanism in the evolution and persistence of a regional genotype(s) / topotype. For example BTV-1 (IND2003/10), and BTV-23 (IND1997/01, IND2004/07, IND2004/08 and IND2004/09) share very closely related versions of Seg-1, -3, -4, -5, -7, -8 and -9. Similarly the majority of the conserved genome segments from Indian isolates of BTV-9 and BTV-16 are also identical or very closely related.

## Conclusions

India has large and diverse populations of ruminants, including camelids and wildlife species. This study significantly increases the number of Indian BTV isolates of different serotypes, for which whole genome-sequence data are available. Full length sequences for each of the different genome segments have supported a detailed analysis of their relationships to viruses from other areas of the world and the incidence of exotic topotypes and reassortment events during strain evolution.

Analysis of Seg-1 and Seg-4 of Indian isolates revealed that these segments are more closely related to Chinese, far eastern and Mediterranean (eastern topotype) strains including vaccine strain of BTV-16. Seg-9 of the majority of Indian isolates with a few exceptions [IND1993/01; IND1994/01] showed very high identities to Greek and Turkish strains of BTV-9e and BTV-16e.

In India two versions of Seg-5 are circulating, one eastern version grouping with strains from China and Europe, and a western version grouping with strains from Nigeria, Libya and Oman. Seg-8 has close association with isolates from Middle East. Seg-10 analysis also showed close relationship of Indian strains to European BTV-9e and BTV-16e strains with the exception of one reassortant strain having a western Seg-10 derived from 10 USA (CA-8). Seg-7 sequences of Indian strains appear to have originated from two different lineages: one close to strains from China, Taiwan and Australia (eastern-topotype 2) with another cluster from China, Australia Turkey and Europe (eastern-topotype 3). Seg-3 sequences [IND2003/08] also fall within eastern group close to Chinese strains.

These analyses indicate the entry of exotic BTV strains from distant geographic regions, including the introduction of western topotypes, particularly western vaccine strains of BTV-2w and BTV-10w from South Africa or the USA respectively. The genome segments of these recent introductions of western topotypes, have been shuffled into otherwise eastern BTV genotypes in the region.The history of severe BTV outbreaks in the Southern states of India in the last decade together with these analyses indicates the importance of continuous monitoring using state of the art diagnosis and surveillance methods. This includes phylogenetic analyses of full genomes of representative isolates to determine the molecular epidemiology, evolutionary history, serotype and topotype of emerging BTV strains. The current tendency to refer to BTV strains simply by serotype, fails to recognise differences that exist in genome segments other than Seg-2 and the significance of topotypel diversity of viruses circulating in the region. The introduction of exotic virus strains/topotypes appears to correlate with a significant increase in the severity of clinical disease [58, 61]. It could also reduce the sensitivity of diagnostic systems and have a significant impact on the efficacy of existing management strategies for this disease. In India, the one western BTV genome segment that we have seen spread very rapidly is Seg-5/NS1. This has happened despite a generally much slower spread of the other western genome segments. Assuming that our data are representative, this might suggests that NS1 has a significant impact on the efficiency of transmission of those reassortants that contain the western NS1 gene, and potentially that NS1 can have a significant impact on virus titre (in cattle and/ or sheep), which potentially could therefore affect virulence, even of otherwise eastern BTV strains. In light of this further work is required to explore, “does the introduction and spread of the western NS1 gene correlate with an increase in virulence of BTV in India and do the more virulent strains in India tend to contain the western NS1 gene?” The cDNA amplification and sequencing strategy that we have developed has provided full length and full genome sequences of multiple BTV isolates from India. It can be used with RNA templates extracted from infected cell culture material (virus isolates) as well as diagnostic samples (e.g. infected blood or spleen) or samples of vector insects. It provides a basis for ongoing molecular epidemiology studies and surveillance of virus topotype, and can also be used with other dsRNA virus isolates (including members of the other 29 *Orbivirus* species). Full characterisation of the virus genome provides information concerning the identity, origins and reassortment of individual genome segments, within novel BTV isolates. This gives a more complete picture of the movement, spread and evolution of outbreak strains, providing a better understanding of ‘risk’, and a basis for decisions concerning the design and implementation of prevention and control strategies.

## Supporting Information

S1 Fig Phylogenetic analysis based on Seg-7/VP7 gene of Indian isolate of BTV with other global isolates. Phylogenetic relationship of full length Seg-7 nucleotide sequences (n = 190) was inferred in MEGA 5 using neighbour-joining method and tested by bootstrapping 1000 replicates. Seg-7 of Indian isolates is depicted with blue dots.
(TIF)

S2 Fig Phylogenetic analysis based on Seg-3/VP3 gene of Indian isolate of BTV with other global isolates. Phylogenetic relationship of full length Seg-3 nucleotide sequences (n = 137) was inferred in MEGA 5 using neighbour-joining method and tested by bootstrapping 1000 replicates. Seg-3 of Indian isolates is depicted with blue dots.
(TIF)

S3 Fig Phylogenetic analysis based on Seg-1/VP1 gene of Indian isolate of BTV with other global isolates. Phylogenetic relationship of full length Seg-1 nucleotide sequences (n = 190) was inferred in MEGA 5 using neighbour-joining method and tested by bootstrapping 1000 replicates Seg-1 of Indian isolates is depicted with blue dots.
(TIF)

S4 Fig Phylogenetic analysis based on Seg-4/VP4 gene of Indian isolate of BTV with other global isolates. Phylogenetic relationship of full length Seg-4 nucleotide sequences (n = 141) was inferred in MEGA 5 using neighbour-joining method and tested by bootstrapping 1000 replicates. Seg-4 of Indian isolates is depicted with blue dots.
(TIF)

S5 Fig Phylogenetic analysis based on Seg-8/NS2 gene of Indian isolate of BTV with other global isolates. Phylogenetic relationship of full length Seg-8 nucleotide sequences (n = 132) was inferred in MEGA 5 using neighbour-joining method and tested by bootstrapping 1000 replicates. Seg-8 of Indian isolates is depicted with blue dots.
(TIF)

S6 Fig Phylogenetic analysis based on Seg-10/NS3 gene of Indian isolate of BTV with other global isolates. Phylogenetic relationship of full length Seg-10 nucleotide sequences (n = 188) was inferred in MEGA 5 using neighbour-joining method and tested by bootstrapping 1000 replicates. Seg-10 of Indian isolates is depicted with blue dots.
(TIF)

S1 Table BTV isolates sequenced as part of this study and accession numbers of genome segments. (XLSX)

S2 Table Percentage identities for BTV Seg-2/VP2 gene. Values for Seg-2 (lower left triangle)/VP2 gene (upper right triangle) for Indian isolates of BTV serotype 1, 2, 3, 9, 10, 12, 16, 21 and 23 are highlighted in different colours.
(XLSX)

S3 Table Percentage identities for BTV Seg-6/VP5 gene. Values for Seg-6 (lower left triangle)/VP5 gene (upper right triangle) for Indian isolates of BTV serotype 1, 2, 3, 9, 10, 12, 16, 21 and 23 are highlighted in different colours.
(XLSX)

S4 Table Percentage identities for BTV Seg-5/NS1 gene. Values for Seg-5 (lower left triangle)/NS1 gene (upper right triangle) for Indian isolates of BTV serotype 1, 2, 3, 9, 10, 12, 16, 21 and 23 are highlighted in different colours.
(XLSX)

S5 Table Percentage identities for BTV Seg-9/VP6 gene. Values for Seg-9 (lower left triangle)/VP6 gene (upper right triangle) for Indian isolates of BTV serotype 1, 2, 3, 9, 10, 12, 16, and 23 are highlighted in different colours.
(XLSX)
