# Supplementary figures and images for: Full-Genome Sequencing as a Basis for Molecular Epidemiology Studies of Bluetongue Virus in India
Source: PLoS One. 2015 Jun 29;10(6):e0131257. doi: 10.1371/journal.pone.0131257 (PMC4488075; doi:10.1371/journal.pone.0131257)

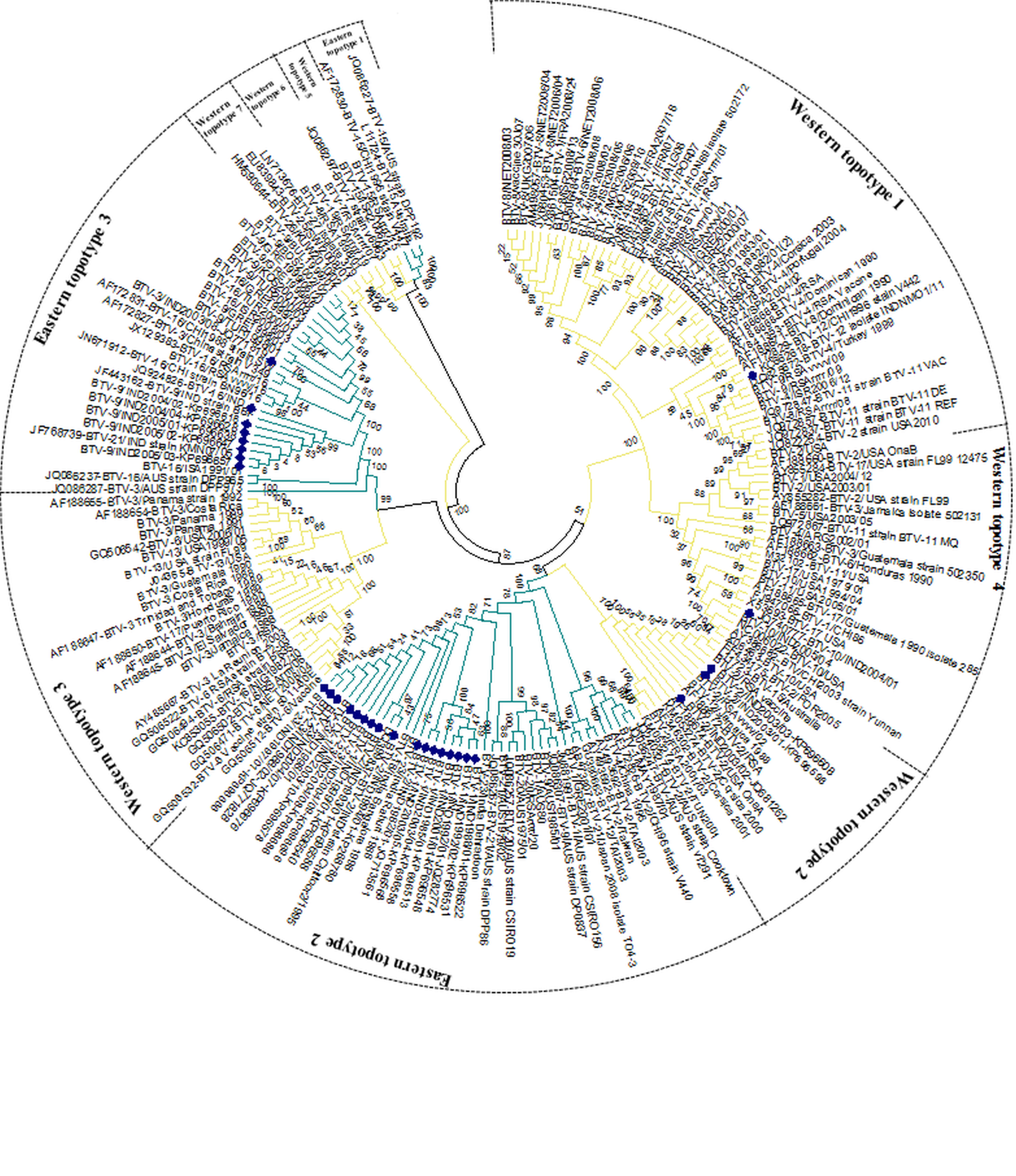

Supplement: S1 Fig — Phylogenetic relationship of full length Seg-7 nucleotide sequences (n = 190) was inferred in MEGA 5 using neighbour-joining method and tested by bootstrapping 1000 replicates. Seg-7 of Indian isolates is depicted with blue dots. (TIF) [file pone.0131257.s001.tif]

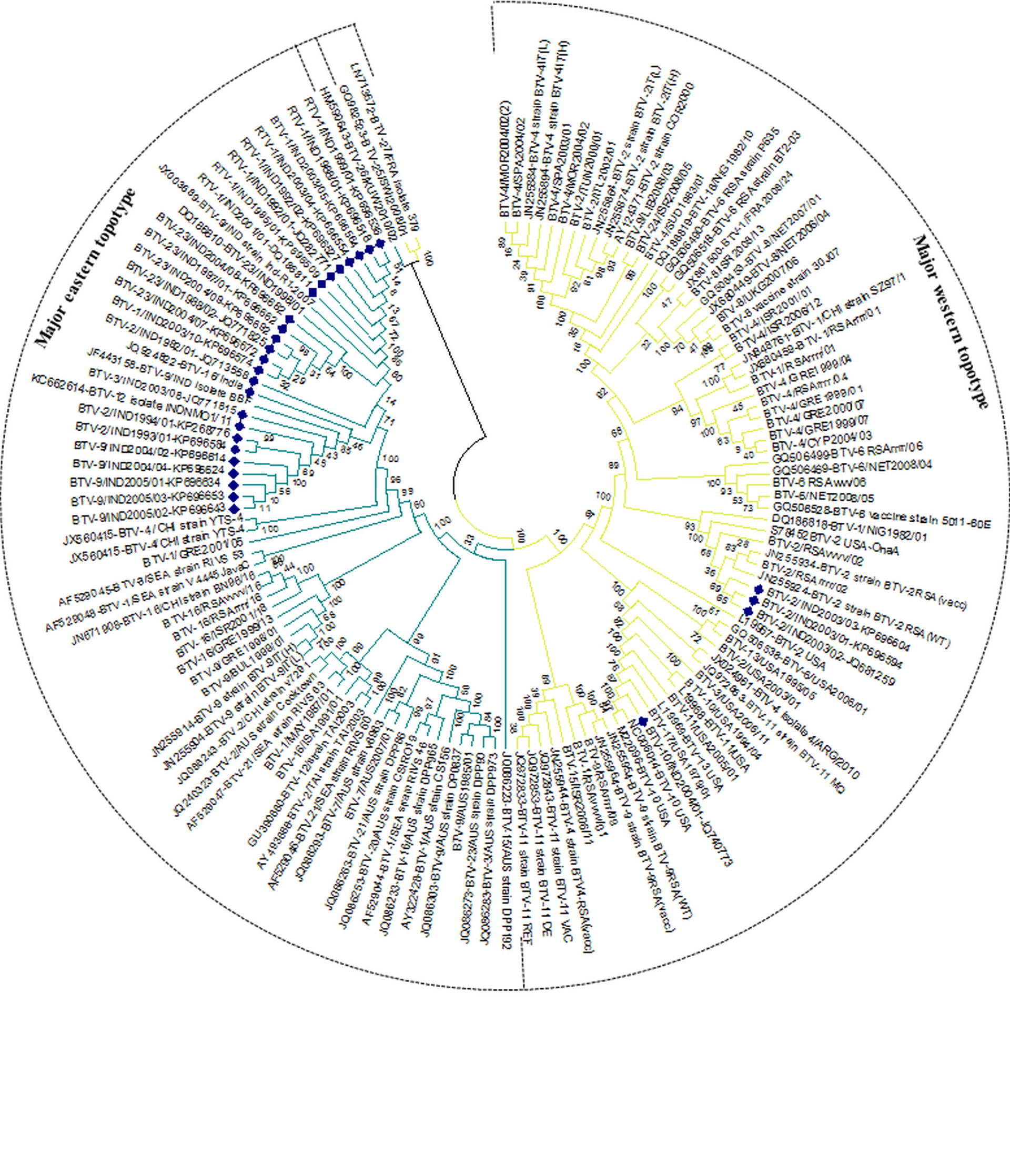

Supplement: S2 Fig — Phylogenetic relationship of full length Seg-3 nucleotide sequences (n = 137) was inferred in MEGA 5 using neighbour-joining method and tested by bootstrapping 1000 replicates. Seg-3 of Indian isolates is depicted with blue dots. (TIF) [file pone.0131257.s002.tif]

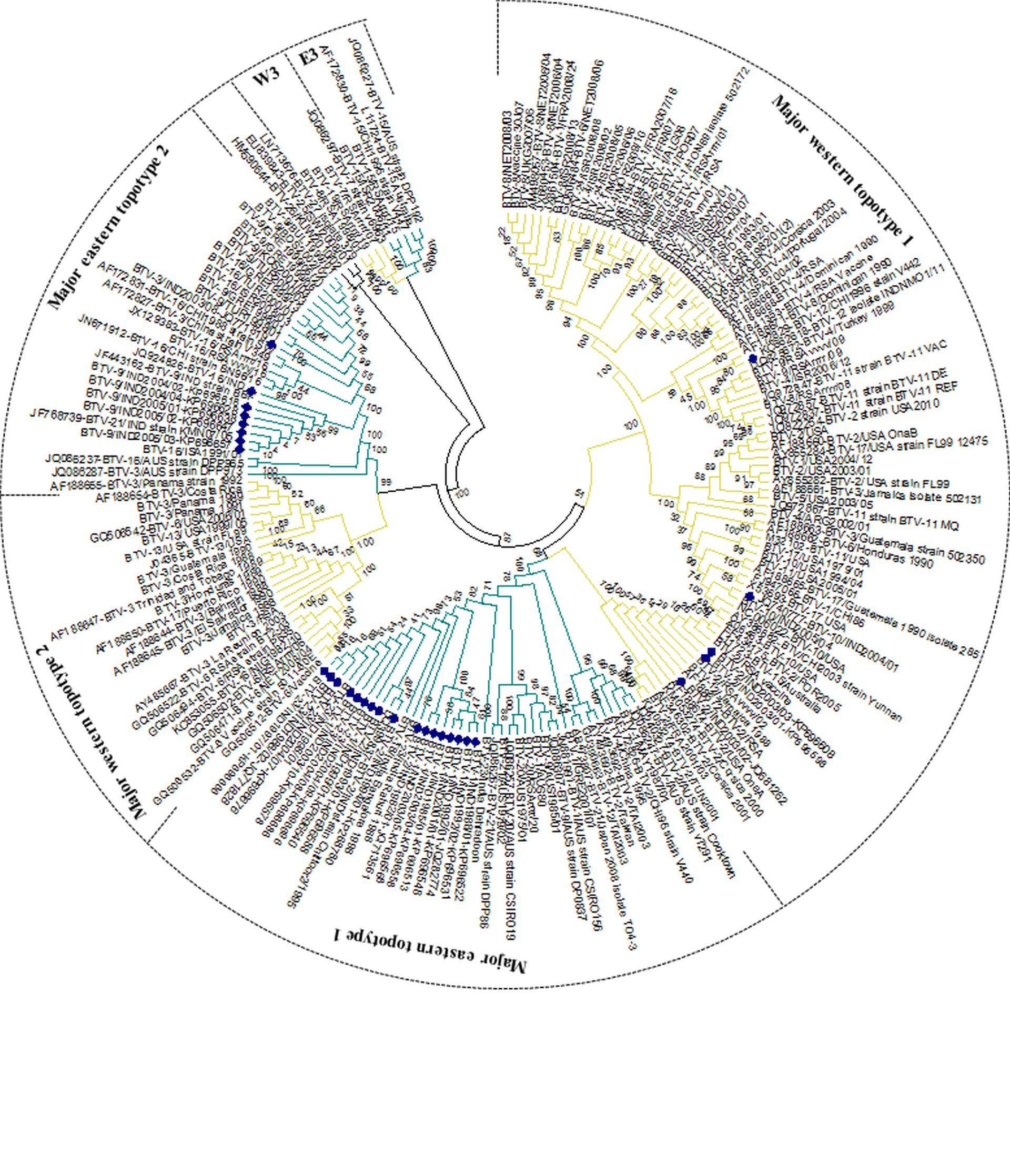

Supplement: S3 Fig — Phylogenetic relationship of full length Seg-1 nucleotide sequences (n = 190) was inferred in MEGA 5 using neighbour-joining method and tested by bootstrapping 1000 replicates Seg-1 of Indian isolates is depicted with blue dots. (TIF) [file pone.0131257.s003.tif]

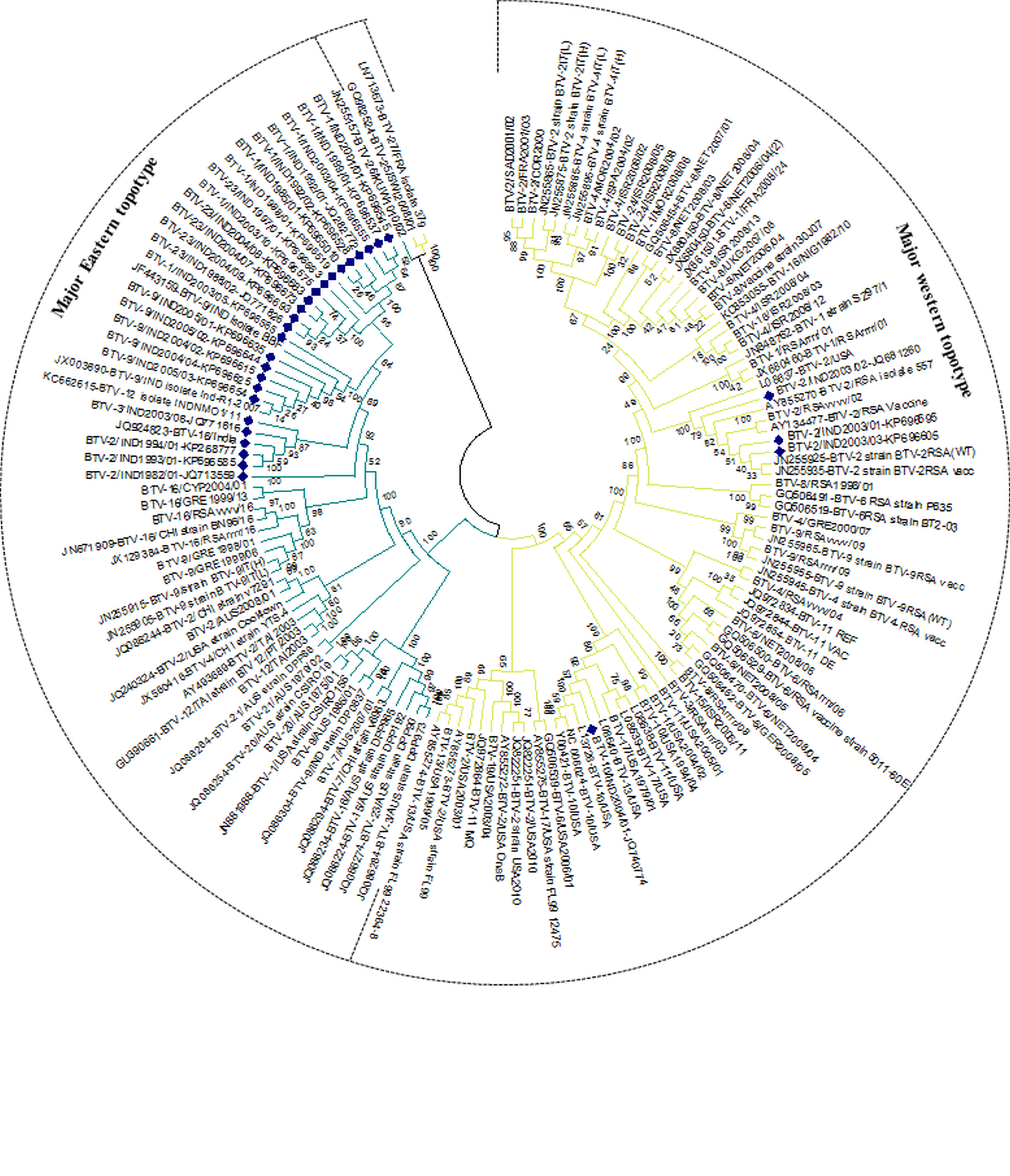

Supplement: S4 Fig — Phylogenetic relationship of full length Seg-4 nucleotide sequences (n = 141) was inferred in MEGA 5 using neighbour-joining method and tested by bootstrapping 1000 replicates. Seg-4 of Indian isolates is depicted with blue dots. (TIF) [file pone.0131257.s004.tif]

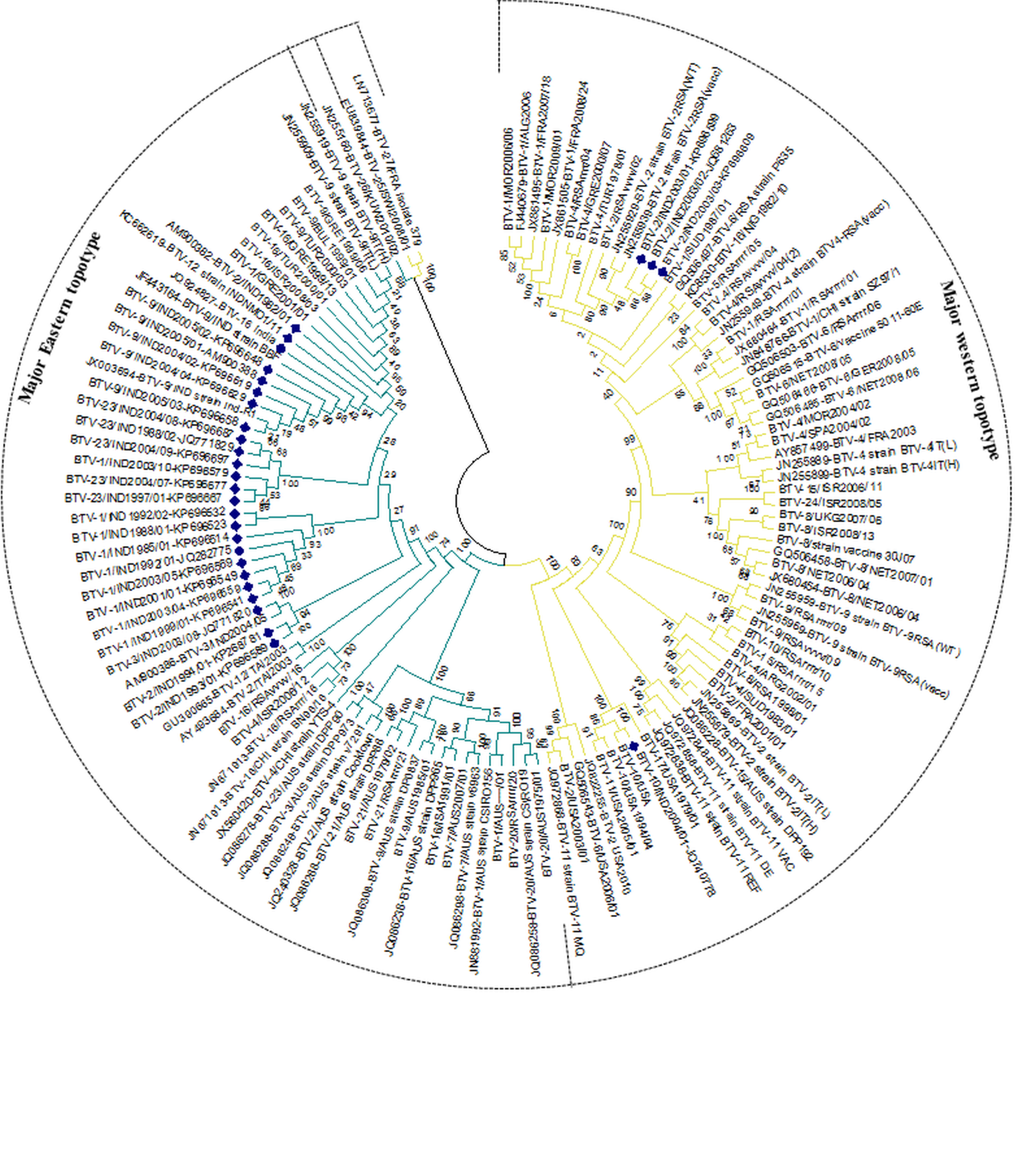

Supplement: S5 Fig — Phylogenetic relationship of full length Seg-8 nucleotide sequences (n = 132) was inferred in MEGA 5 using neighbour-joining method and tested by bootstrapping 1000 replicates. Seg-8 of Indian isolates is depicted with blue dots. (TIF) [file pone.0131257.s005.tif]

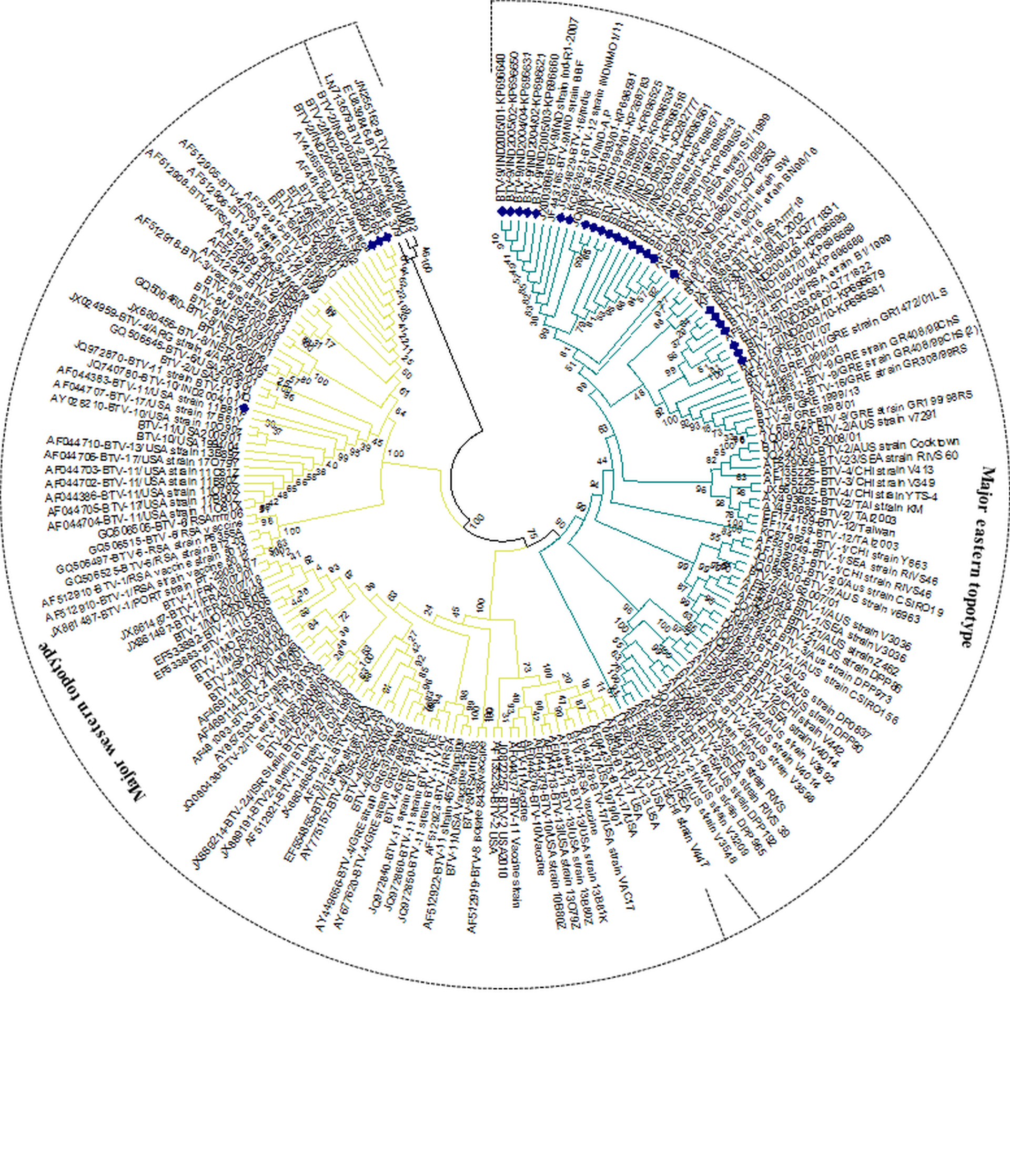

Supplement: S6 Fig — Phylogenetic relationship of full length Seg-10 nucleotide sequences (n = 188) was inferred in MEGA 5 using neighbour-joining method and tested by bootstrapping 1000 replicates. Seg-10 of Indian isolates is depicted with blue dots. (TIF) [file pone.0131257.s006.tif]
